# Determining phase transitions of layered oxides via electrochemical and crystallographic analysis

**DOI:** 10.1080/14686996.2020.1814116

**Published:** 2020-09-15

**Authors:** Katja Fröhlich, Isaac Abrahams, Marcus Jahn

**Affiliations:** a Electric Drive Technology, AIT Austrian Institute of Technology GmbH, Vienna, Austria; b School of Biological and Chemical Sciences, Queen Mary University of London, London, UK

**Keywords:** Lithium nickel manganese cobalt oxide, NMC, crystallographic analysis, chemical diffusion coefficient, phase transition, GITT, 207 Fuel cells, Batteries, Super capacitors

## Abstract

The chemical diffusion coefficient in LiNi_1/3_Mn_1/3_Co_1/3_O_2_ was determined via the galvanostatic intermittent titration technique in the voltage range 3 to 4.2 V. Calculated diffusion coefficients in these layered oxide cathodes during charging and discharging reach a minimum at the open-circuit voltage of 3.8 V and 3.7 V vs. Li/Li^+^, respectively. The observed minima of the chemical diffusion coefficients indicate a phase transition in this voltage range. The unit cell parameters of LiNi_1/3_Mn_1/3_Co_1/3_O_2_ cathodes were determined at different lithiation states using *ex situ* crystallographic analysis. It was shown that the unit cell parameter variation correlates well with the observed values for chemical diffusion in NMC cathodes; with a notable change in absolute values in the same voltage range. We relate the observed variation in unit cell parameters to the nickel conversion into the trivalent state, which is Jahn-Teller active, and to the re-arrangement of lithium ions and vacancies.

## Graphical Abstract

**Figure:**
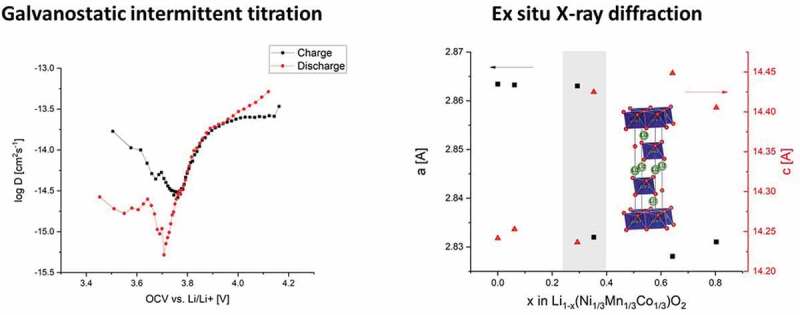

## 1. Introduction

Li-ion batteries are the leading electrochemical storage systems for both small and large-scale applications, from mobile phones up to electric vehicles (EVs). Their energy density, among other important factors, is influenced by the cathode material, which is not only the main contributor to the overall cost, but also the major determining factor for the cell capacity [1,2].

Mixed layered oxide cathodes are one of the most promising candidates to meet future requirements for EV applications. LiNi_1/3_Mn_1/3_Co_1/3_O_2_ (NMC), as the most common representative, has already been studied extensively [3,4], including work aimed at improving the materials’ properties by employing strategies such as doping and coating [5–7]. Among other cathode materials such as LiNi_0.5_Mn_1.5_O_4_ (LNMO) spinels [8], NMC has already found its way into commercialization within the automotive sector, with Nissan being a prominent adopter of this material from the start.

**Figure 1.**
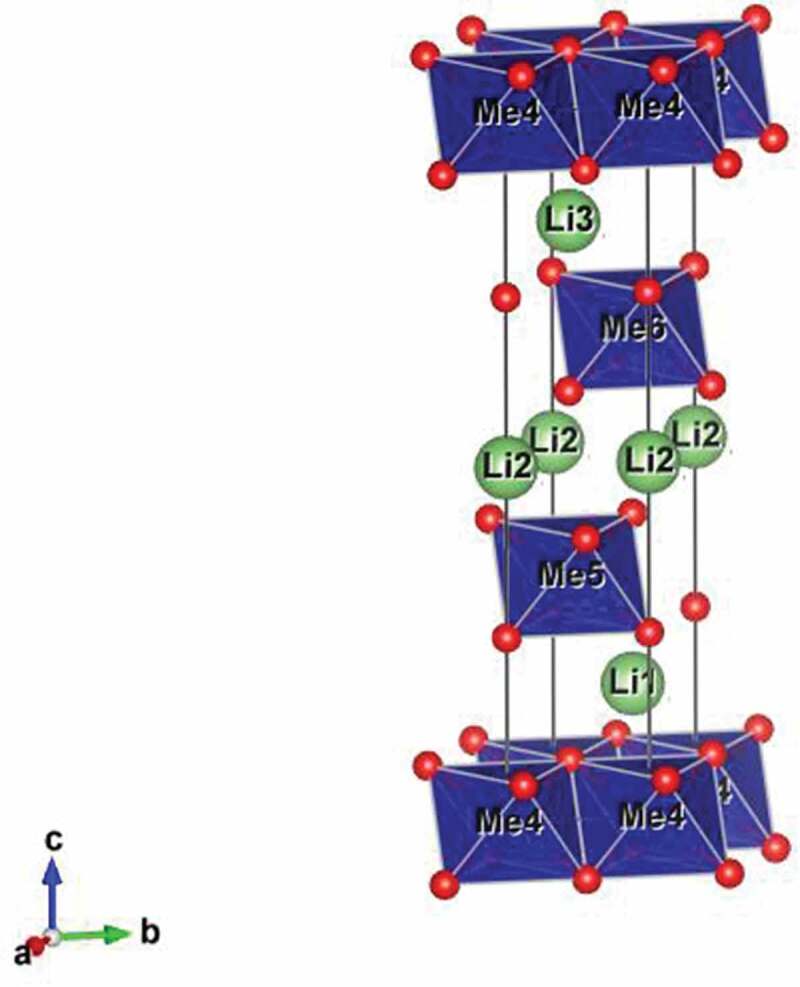
Structure of LiMeO_2_, showing metal (Me) octahedra (blue) and positions of oxygen (red) and lithium (green) atoms.

LiNi_1/3_Mn_1/3_Co_1/3_O_2_ (NMC) exhibits the delafossite (NaCrS_2_) structure in space group *R*-3 *m* and is based on a cubic close packed oxide ion array with transition metal cations occupying all the octahedral sites in alternate layers. The Li^+^ cations are located in the remaining octahedral sites between the transition metal oxide layers (Figure 1). The layered structure thus provides two-dimensional diffusion pathways for Li^+^ ion insertion and extraction during discharging and charging, respectively.

It is widely agreed that the oxidation states of the framework cations are Ni^2+^, Mn^4+^ and Co^3+^ in the fully lithiated, discharged cathode material LiNi_1/3_Mn_1/3_Co_1/3_O_2_ [9]. However, the literature is inconsistent regarding the charge compensation reaction. Even though most agree that charge compensation during extraction of Li^+^ is related to Ni oxidation, *i.e*. Ni^2+^ → Ni^3+^ at lower potentials and Ni^3+^ → Ni^4+^ at higher potentials, some results indicate that the Ni^3+^/Ni^4+^ redox pair is not active in the applied cycling range [10]. This would explain the irreversibility of nickel-rich NMC cathode materials, which naturally exhibit a higher amount of Ni^3+^, and therefore are only stable during cycling at lower cut-off voltages, depending on the overall amount of Ni in the structure [11,12].

In addition, the role of Co is not fully clarified. Early work based on a combined experimental and theoretical study by Ceder’s group states that Co oxidation from 3+ to 4+ occurs only in highly delithiated NMC [13]; whereas recent work suggests that the Co^3+^/Co^4+^ redox couple is active over the whole cycling range even at higher lithiated states [14].

The inconsistency of the published data regarding different NMC compositions reveals that a deeper understanding of structural changes in combination with charge compensation during cycling is needed to improve future NMC cathode materials for automotive applications.

The lithium ion diffusion coefficient (*D*_Li+_) is a key indicator of charge kinetics, as the mobility of Li^+^ ions in electrode structures is crucial for efficient charging/discharging of battery cells. The crystal structure of the corresponding electrode materials therefore determines the reaction kinetics.

Different methods have been applied for the determination of the diffusion coefficient in NMC, such as cyclic voltammetry (CV) [15,16], electrochemical impedance spectroscopy (EIS) [17–19] and galvanostatic intermittent titration technique (GITT) measurements [20–23]. The observed absolute values in previously published work differ considerably, ranging from 10^−10^ to 10^−19^ cm^2^ s^−1^.

The reason for the wide variation in absolute values lies in the differences between the corresponding methods, electrode fabrication and porosity, as well as the estimation of several parameters needed to calculate the diffusion coefficient. In addition, the influence of the experimental setup is often underestimated, especially for impedance measurements. The porosity of the electrodes in combination with small size, as is the case in most studies, can lead to reproducibility problems in terms of absolute values of chemical diffusion coefficients. Furthermore, to calculate the diffusion coefficient, one must know the effective reaction surface area, which is not directly accessible and therefore estimated, e.g. via gas adsorption measurements.

Among these methods for analysing the diffusion behaviour in cathode materials, GITT serves as a powerful, tool linking charge kinetics and thermodynamics in insertion compounds. Even though the method has some general assumptions, such as one-dimensional Ficksian diffusion, uniform particle size and shape, concentration dependence and homogeneous surfaces, it is useful for analysing phase transformations and structural changes due to its combination of steady and transient states during the measurement. To interpret the calculated diffusion coefficient curves for charging and discharging over the cycling range, parallel analysis of the crystal structure variation can help in understanding the variation of the chemical diffusion coefficient.

The description of structural changes and their relationship to the electrochemical properties is seen as an essential part of research on next generation host matrices for Li^+^-ion batteries [7]. Several studies have focused on structure variation during cycling involving *in situ* and *in operando* measurements [20,24–28]. Problems such as the large incoherent scattering of hydrogen for neutron diffraction measurements, analysis of the diffraction from multiple components, the design and construction of specially adapted cells, including electronically insulating windows and/or equipment, make these studies comparatively difficult.

*Ex situ* diffraction investigations on NMC and other insertion compounds also have been performed [29–31], sometimes in combination with other characterisation techniques or *in situ* methods. In the case of NMC, trends in the lattice parameters at different lithiation states have been identified. Nevertheless, none of the aforementioned studies linked the unit cell parameter variation to the OCV, but rather to *x* in Li_1-*x*_Ni_1/3_Mn_1/3_Co_1/3_O_2_.

The aim of the present work is to clarify the phase behaviour of NMC during cycling. To achieve this, GITT has been applied to determine the open-circuit voltage (OCV) at different lithiation states of NMC and to calculate the chemical diffusion coefficient, $\tilde{D}$, over the whole cycling range. It is found that $\tilde{D}$ exhibits minima at the oxidation/reduction peaks during charging and discharging. This type of behaviour is commonly related to phase transitions or structural re-arrangements.

To prove structural re-arrangement, *ex situ* X-ray powder diffraction (XRD) measurements of NMC cathodes were carried out using adapted coin cells, and the unit cell parameters were fitted via Rietveld analysis. In this way, the lattice parameter variation was linked with the OCV curve obtained from the GITT measurement, allowing for conclusions regarding phase transformation during charge compensation.

## 2. Experimental details

### 2.1. Synthesis

Lithium nickel manganese cobalt oxide, LiNi_1/3_Mn_1/3_Co_1/3_O_2_ (NMC), was synthesized via a co-precipitation route. Aqueous solutions (0.2 mol dm^−3^) of Mn^2+^, Ni^2+^ and Co^2+^ were prepared from Mn(NO_3_)_2_∙4H_2_O (Merck, 98%), Ni(NO_3_)_2_∙6H_2_O (Merck, 99%) and Co(NO_3_)_2_∙6H_2_O (Alfa Aesar, 98%), respectively, to give the desired stoichiometry. The solutions were then mixed, and concentrated ammonia was added in a 6:1 ratio with respect to the total cation content. Following this, NaOH (2 mol dm^−3^) was successively added in a dropwise manner to the solution to initiate the precipitation reaction. The total amount of NaOH used was calculated to give a 2:1 ratio with respect to the total cation content. The resultant precipitate was then filtered and washed several times with deionized water before drying for 14 h at 120°C in air. The dried powder was then milled with excess (3 mol%) LiOH∙H_2_O (Alfa Aesar, 98%) in a planetary ball mill and then placed in a zirconia boat at 500°C for 5 h under flowing synthetic air. After the heat treatment, the sample was cooled to room temperature, ground in an agate mortar then placed in a zirconia boat at 900°C for 10 h in flowing synthetic air. All heating and cooling measurements were carried out at a rate of 5°C min^−1^. Elemental composition was determined by inductively coupled plasma optical emission spectroscopy (ICP-OES; PerkinElmer OPTIMA 7300 DV, Massachusetts, USA) and the data analysed using WinLab32 for ICP. The powders were chemically pulped using a concentrated nitric and hydrochloric acid mixture under constant heating. Argon was used as the analysis gas and the equipment was calibrated prior to the analysis.

### 2.2. Electrochemical measurements

Cathodes were prepared with a 85:8:2:5 mass ratio of synthesized NMC as active material, carbon black – Super P (Timcal), SFG6L graphite (MTI) and PVDF binder (MTI) and coated onto an aluminium current collector to achieve a final coating thickness of 50 µm. The dried and calendared cathodes were assembled into 2032 type coin cells for half-cell measurements against metallic lithium. 1 M LiPF_6_ in EC/DMC 1:1 (w/w) (LP30, BASF) was used as electrolyte. All electrochemical tests were performed using a Maccor Series 4000 battery tester (Oklahoma, USA).

Galvanostatic intermittent titration technique (GITT) measurements were performed to determine the chemical diffusion coefficient in the NMC cathodes over the whole cycling range from 3 to 4.2 V. After two formation cycles at 0.1 C, current pulses of 600 s at 0.1 C were applied and the relaxation time of the cell after each pulse was set to 2400 s. The open-circuit voltage (OCV) was directly derived from the experiment, whereas the chemical diffusion coefficient in the cathode was calculated accordingly as first described by Weppner and Huggins [32], based on Fick’s second law. If the time scale of the pulse is considerably smaller than the length of the electrode divided by the chemical diffusion coefficient, $\tilde{D}$ can be calculated via:

(1) $$\tilde{D}=\;\frac{4}{\pi }\;{\left(\frac{m_{B}\;V_{m}}{M_{B}\;S}\right)}^{2}{\left(\frac{\Delta U_{OCV}}{\;\tau \left(\frac{dU}{d\sqrt{t}}\right)}\right)}^{2}$$

Where τ represents the duration of the pulse, *m_B_* the active mass of the electrode, *V_M_* the molar volume of the active material, *M_B_* the molecular weight, and *S* the active specific surface area of the electrode. *ΔU_OCV_* represents the open-circuit voltage difference before and after the pulse; whereas *ΔU_t_* is the actual change of the voltage during the pulse, neglecting the IR-drop.

Cyclic voltammetry (CV) measurements were performed prior to the crystallographic analysis with a scan rate of 0.1 mV s^−1^ up to 4.5 V. Cycling was stopped at different lithiation states and cells were assembled and disassembled under inert atmosphere in an argon filled glove box (Mbraun, Germany). After disassembly, the cathodes were washed with dimethyl carbonate (BASF, Germany) to remove residual lithium salts. For the powder-XRD measurements, the dried cathodes were sealed in a specially adapted coin cell covered with Mylar film to prevent air exposure during the measurement period (see Fig S1 in supplementary information).

Powder X-ray diffraction data were recorded on a PANAlytical X’Pert Pro diffractometer (United Kingdom), with an X’Celerator detector in the 2θ range 5–120° with a step width of 0.0167° and an effective count time of 200 s per step. Ni filtered Cu-Kα (λ = 1.5418 Å) radiation was used with the sample in flat plate θ/θ geometry. Structural analysis was carried out using the GSAS (General Structure Analysis System) package [33] with the EXPGUI interface [34]. In all cases, a polynomial background, scale factor, zero point, absorption correction, preferred orientation, coefficients for the peak shape function and cell parameters were refined. The structure of LiNi_1/3_Mn_1/3_Co_1/3_O_2_ presented by Nazar et al. [35] was used as a starting model (see Table S1, Supplementary Information).

Due to the number of distinct phases present in the cathode samples, reference patterns of the graphite and aluminium current collector used were recorded and fitted by Rietveld analysis. The results obtained were used in the Rietveld analysis of the cathode samples as secondary phases. In these multiphase refinements the structural parameters of the secondary phases were fixed. The actual lithiation state of the NMC samples was calculated from the specific capacity of each cathode material. These values were used in the structural model for the Rietveld refinement.

## 3. Results and discussion

### 3.1. Cyclic voltammetry (CV)

**Figure 2.**
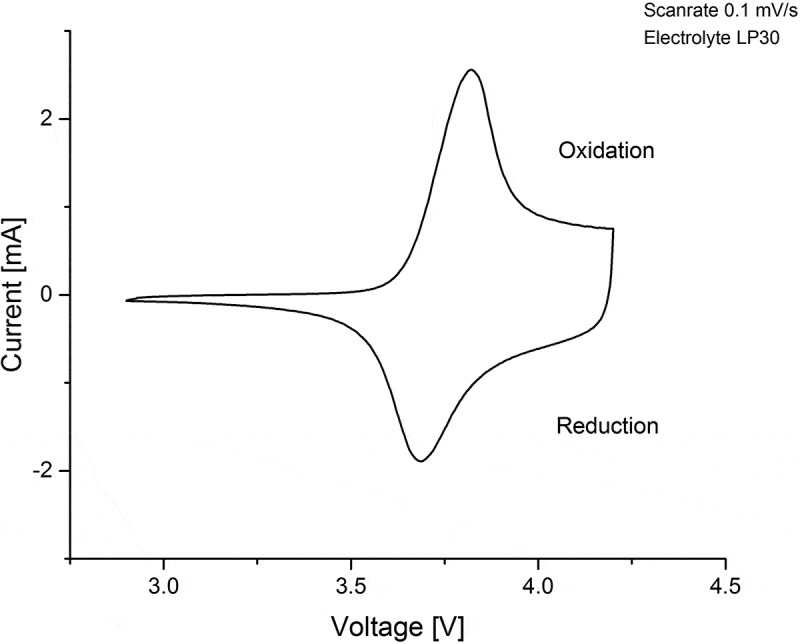
Cyclic voltammogram of NMC. The sharp peaks correspond to the redox couples of the charge compensation reaction.

Figure 2: Cyclic voltammogram of NMC. The sharp peaks correspond to the redox couples of the charge compensation reaction. shows a cyclic voltammogram at a scan rate of 0.1 mV s^−1^. The polarisation between the oxidation peak at 3.8 V and the reduction peak at 3.7 V is 100 mV. Only one anodic and one cathodic peak are present. The absence of a peak at 3 V implies that no Mn^3+^ was present in the synthesized NMC cathode structure [36]. The oxidation and reduction peaks correspond to the charge compensation reaction during Li^+^ extraction and insertion, whereas the small polarisation reflects the reversibility of this process.

### 3.2. Galvanostatic intermittent titration technique (GITT)

**Figure 3.**
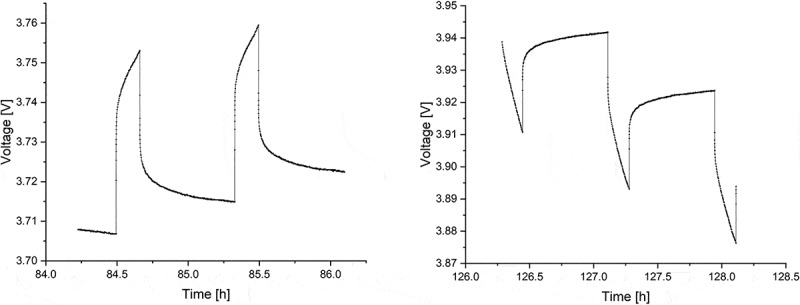
Measured voltage curves for the 10th and 11th charge (left) and discharge (right) pulses of synthesized NMC from the GITT measurement.

Figure 3: shows the 10th and 11th voltage profiles of charge and discharge pulses during GITT measurements, respectively (full voltage range profiles are given in Fig. S2 in the supplementary information).

The voltage changes during the pulses for both charging and discharging show linear behaviour, such that the formula for $\tilde{D}$ simplifies to:

(2) $$\tilde{D}=\;\frac{4}{\pi \;.\tau }\;{\left(\frac{m_{B}\;V_{m}}{M_{B}\;S}\right)}^{2}{\left(\frac{\Delta U_{OCV}}{U_{t}}\right)}^{2}$$

**Figure 4.**
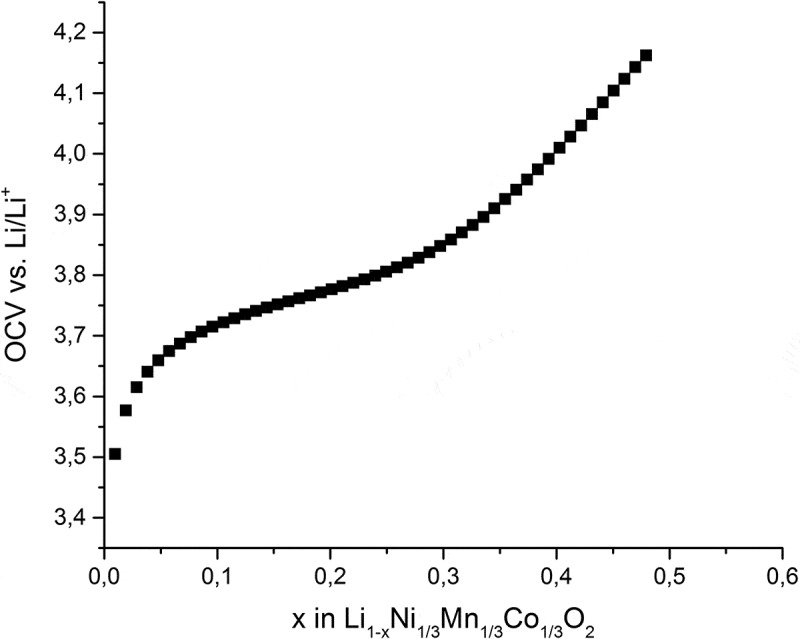
Open-circuit voltage (OCV) of NMC at different lithiation states.

The open-circuit voltage (OCV) as obtained from GITT measurements over the cycling range for the charging case is presented in Figure 4. It shows the typical plateau region of NMC cathodes between 3.7 and 3.85 V. In this experiment, approximately half of the lithium ions were reversibly extracted from the structure according to the measured cell capacity.

**Figure 5.**
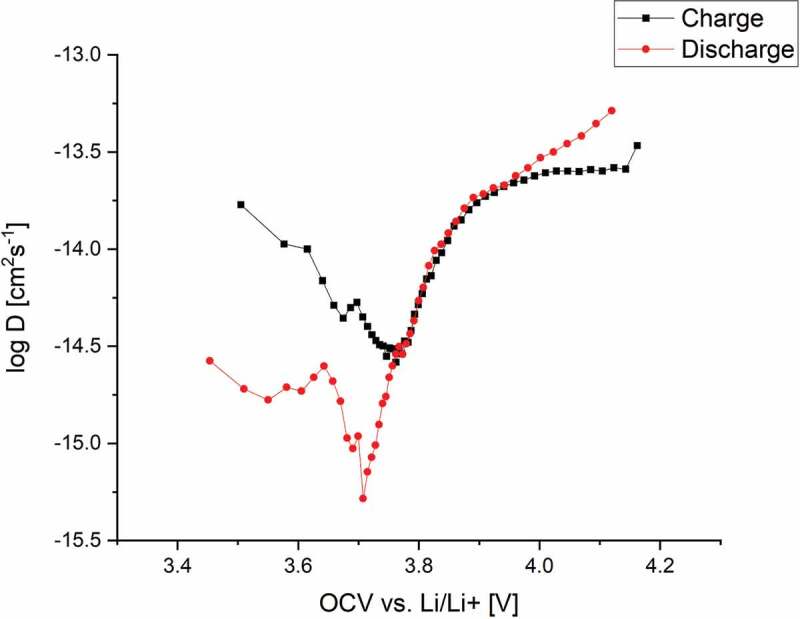
Calculated chemical diffusion coefficient in NMC over the OCV during charging and discharging.

Figure 5 displays the chemical diffusion coefficient $\tilde{D}$ for both charging and discharging over the measured open-circuit voltage. Both curves show a V-type shape, where $\tilde{D}$ on discharging reaches a minimum at the OCV of 3.7 V, whereas the charge diffusion has its minimum at 3.8 V.

The value of $\tilde{D}$ for charging is significantly higher compared to that for discharging up to 3.8 V. Whilst the lithium ion diffusion coefficient *D*_Li+_ would be expected to show similar values for charging and discharging, $\tilde{D}$ contains several components and these would not necessarily be expected to show the same behaviour. The chemical diffusion coefficient reflects all species and, especially in combination with phase transformations as observed for other Li^+^ insertion materials [37,38], can differ for charge and discharge cases as observed from the presented GITT measurements. The calculated thermodynamic factor as well as the component diffusion coefficient *D*_Li+_ are presented in the supplementary information in Fig S3.

The calculation of $\tilde{D}$ requires the estimation of the reaction surface area of the corresponding electrode. Even though this leads to an unknown scaling factor and wide range of published absolute values, the overall tendencies of the diffusion coefficients for insertion and extraction of lithium ions are evident. The reaction area is solely incorporated as a linear parameter and therefore slightly shifts the curves over the measured voltage range.

Local diffusion coefficient minima observed by other groups [4,37–39] are usually associated with structural changes such as phase transitions in combination with the charge compensation reaction during Li^+^ insertion/extraction. In the case of LiNi_1/3_Mn_1/3_Co_1/3_O_2_, little detail is known regarding possible phase transitions or structural changes over the cycling range used in the present study. Significant structural changes in NMC type cathodes usually occur at much higher voltages above 4.5 V [40–42]. Only Shaju’s group stated that a phase transition takes place in this voltage range, which must be reversible as no significant decay in capacity is visible [21]. The good cyclability of NMC; even at 5 C over 200 cycles [43], or when new production routes are applied [2]; indicates the reversible nature of the structural rearrangement.

The oxidation/reduction peaks from the cyclic voltammetry measurements correlate well with the variation of the diffusion coefficients for the charge and discharge cases, where the minima of $\tilde{D}$ were observed at the peak positions and all nickel ions are supposed to be in the 3+ state. The Ni^3+^ ion is Jahn-Teller active and can therefore trigger distortion or lead to phase transformation in combination with Li^+^ ion vacancy ordering such as in LiNiO_2_ [44].

### 3.3. Ex situ XRD

To investigate the structural rearrangement, crystallographic analysis of cycled cathodes at different lithiation states in the same voltage range was performed. The corresponding X-ray diffraction profiles are given in the supplementary information as Fig. S4. The fitted XRD pattern of the synthesised powder (Fig. S5, with corresponding crystal and refinement data in Table S2 and refined parameters in Table S3) confirms the phase purity of the synthesized NMC, while ICP analysis confirms the calculated stoichiometry (Li 6.9 ± 0.1 wt% Ni 20.2 ± 0.2 wt%, Mn 19.3% ± 0.1 wt% and Co 20.1% ± 0.2 wt%).

**Figure 6.**
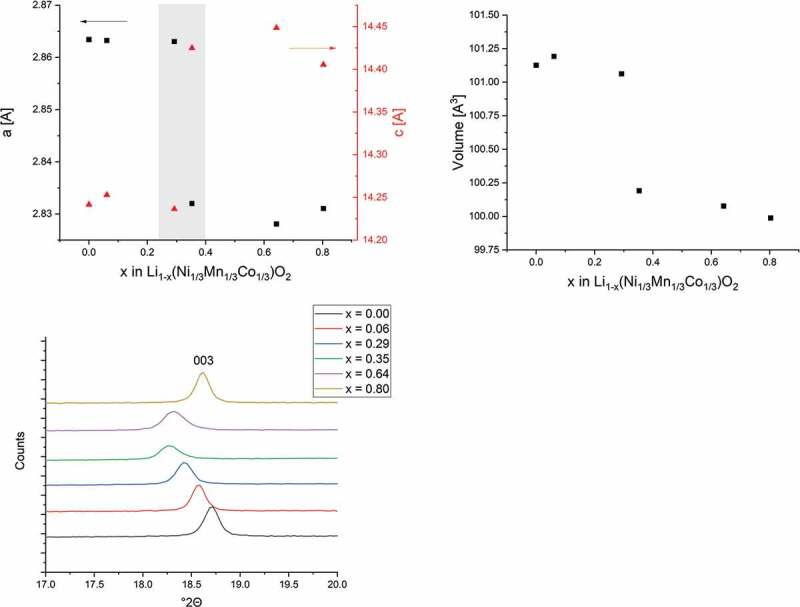
Unit cell parameter variation of NMC cathodes, derived from Rietveld refinement of *ex situ* XRD patterns. [a] *a* and *c*-axis, [b] unit cell volume, both vs. open-circuit voltage (estimated standard deviations are smaller than the symbols used) and [c] detail of diffraction profiles for NMC cathodes showing the characteristic [003] peak.

Figure 6 shows the variation of the unit cell parameters and the *c*/*a*-ratio with open-circuit voltage. The *a*-axis (representing the intra-slab distance) shows a small decreasing trend with increasing OCV up to 3.8 V, with a sharp decrease between 3.8–3.9 V. During charging of the cell, the amount of Li^+^ in the structure decreases, and charge compensation in the NMC structure occurs via the oxidation of Ni^2+^ ions [45]. The decreasing *a*-parameter is related to the smaller ionic radii of Ni^3+^ and Ni^4+^ compared to Ni^2+^ with values of 0.60 Å (HS), 0.48 Å and 0.69 Å, respectively, for the ions in six-coordinate geometry [46]. In contrast, the *c*-axis (representing the inter-slab distance, where the lithium ions are intercalated) shows an increase between 3.8 and 3.9 V.

The extraction of Li^+^ ions from a host structure would usually be associated with a decrease of the interlayer-spacing and therefore the observed increase is unexpected. Existing arguments regarding electrical repulsion between the MeO_6_-layers are valuable [47], but would likely show a steady increase rather than a sudden change as observed. The present data indicate a sudden structural re-arrangement of the lithium ions and vacancies between the layers, leading to an abrupt decrease of $\tilde{D}$ just before the observed unit cell parameter change.

The variation in unit cell volume is dominated by the change in the *a*-axis, with a sharp decrease in the range 3.8–3.9 V. The overall unit cell volume changes only slightly by ~ 1%, which is favourable for electrode materials to avoid cracking, as can happen in Li-excess NMC and at higher cut-off voltages [48]. Similar trends were observed by other groups in *ex situ* XRD studies on cycled NMC and other cathode materials, but less pronounced in terms of sudden changes in lattice parameters than seen here [29–31]. In addition, previous studies have focused on variation of diffusion coefficient with composition rather than the OCV of the cell and therefore, the abrupt changes in lattice parameters are less visible.

Significant unit cell volume decay has been reported for various layered oxide cathodes compounds, such as NMC811 [12], LiNiO_2_ [49], and LiCoO_2_ only at higher potentials [50], where it was mainly related to the irreversible phase transition between the two hexagonal phases H2 → H3. The presented data suggest structural re-arrangement at *x* = 0.64. In pure LiCoO_2_, only about half of the Li^+^ ions can be reversibly extracted, due to lithium/vacancy ordering, which leads to a structural transformation from the hexagonal to the monoclinic phase [51]. Reversibility is lost in LiCoO_2_ above this state, whereas NMC can be reversibly cycled up to 4.3 V against Li/Li^+^; corresponding to ~ 2/3 of the total lithium content.

The characteristic change of the unit cell parameters in the range of 3.8–3.9 V is correlated to the determined minimum in $\tilde{D}$. In this voltage range, Li^+^ ion diffusion is lower, and the kinetics for both lithium ion extraction and insertion, reach their minimum.

NMC can be described as a solid solution of the parent lithiated transition metal oxides LiNiO_2_, LiCoO_2_ and LiMnO_2_ and therefore exhibits characteristics of the corresponding phases [52]. Cobalt oxidation in LiCoO_2_-rich regions of NMC, where only half of Li^+^ can reversibly be extracted, is frustrated where the structural re-arrangement occurs and the calculated $\tilde{D}$ reaches its minimum. Reversible crystal structure transformation between the hexagonal and monoclinic phases of NMC during the charge compensation reaction have previously identified for LiNiO_2_ [49,53,54]. The observed phase change is related to the charge compensation reaction and therefore reduces Li^+^ ion diffusion at the oxidation/reduction potentials of both Ni^2+^ and Ni^3+^ with some contribution of Co^3+^/Co^4+^ redox pairs.

The coincidence of the presented data, the typical plateau region during cycling, the observed minimum of $\tilde{D}$ for both charge and discharge, which correlate with the oxidation/reduction peaks as well as the abrupt change in unit cell parameters, lead to the conclusion that a phase transition and/or reversible phase separation occurs at an OCV of approximately 3.8 V, which might be induced by the Jahn-Teller active Ni^3+^-ions.

## 4. Conclusions

The variation of the chemical diffusion coefficient and crystal structure in LiNi_1/3_Mn_1/3_Co_1/3_O_2_ was measured during electrochemical cycling against Li/Li^+^ in 2032 coin cells over the OCV range 3.0 to 4.2 V. It was shown that the $\tilde{D}$ of the NMC cathodes exhibits minima at 3.8 V and 3.7 V for charging and discharging, respectively. The obtained minima are in the same region as the oxidation/reduction peaks from cyclic voltammetric measurements, indicating that structural changes related to phase transformation occur during the charge compensation reaction via the Ni^2+/3+^ redox couple.

These structural changes are associated with abrupt changes in lattice parameters, which occur just after the minima in $\tilde{D}$. These are suggested to be due to sudden changes in the Li^+^-ion/vacancy distribution at critical OCV values. Thus, NMC appears to undergo a phase transition and/or phase separation during cycling in the range 3.0 to 4.2 V, which is associated with the charge compensation reaction during Li^+^ extraction and insertion. These changes during the redox process limit the Li^+^-ion diffusion in the cathode material and must therefore be fully understood before work towards less stable Ni-rich and low-Co NMC cathode materials continues.

The simple experimental setup used here could be easily applied to many electrochemical measurements under realistic conditions. The presented experimental methodology represents a relatively facile way to study structural changes at higher cut-off voltages and could help in understanding how to stabilise future host structures at higher voltages.

## Supplementary Material

Supplemental Material

## References

[1] Zubi G, Dufo-López R, Carvalho M, et al. The lithium-ion battery: state of the art and future perspectives. Renewable Sustainable Energy Rev. 2018;89:(March):292–308.

[2] Fröhlich K, Legotin E, Bärhold F, et al. New large-scale production route for synthesis of lithium nickel manganese cobalt oxide. J Solid State Electrochem. 2017;21(12):3403–3410.

[3] Ceder G. Van Der Ven A. Phase diagrams of lithium transition metal oxides: investigations from first principles. Electrochim Acta. 1999;45(1):131–150.

[4] Ohzuku T, Makimura Y. Layered lithium insertion material of LiCo_1/3_Ni_1/3_Mn_1/3_O_2_ for lithium-ion batteries. Chem Lett. 2001;7(7):642–643.

[5] Xu H, Ye X, Xiao C, et al. Synthesis and electrochemical performance of Mg-Doped Li(Ni _1/3_ Co _1/3_ Mn _1/3_) _1–x_ Mg _x_ O _2_ cathode material for lithium-ion battery. Synth React Inorganic Met Nano-Metal Chem. 2015 2;45(2):234–237.

[6] Li Y, Li Y, Zhong S, et al. Synthesis and electrochemical properties of Y-Doped LiNi_1/3_Mn_1/3_Co_1/3_O_2_ cathode materials for li-ion battery. Integr Ferroelectr. 2011;127(1):150–156.

[7] Wang Y, Zhang W, Chen L, et al. Quantitative description on structure–property relationships of Li-ion battery materials for high-throughput computations. Sci Technol Adv Mater. 2017;18(1):134–146. 28458737 10.1080/14686996.2016.1277503 PMC5402746

[8] Jehnichen P, Wedlich K, Korte C Degradation of high-voltage cathodes for advanced lithium-ion batteries – differential capacity study on differently balanced cells. 2018

[9] Erickson EM, Schipper F, Penki TR, et al. Review—recent advances and remaining challenges for lithium ion battery cathodes. J Electrochem Soc. 2017;164(1):A6341–8.

[10] Miao S, Kocher M, Rez P, et al. Local electronic structure of layered LixNi_0.5_Mn_0.5_O_2_ and Li_x_Ni_1/3_Mn_1/3_Co_1/3_O_2_. J Phys Chem B. 2005;109(49):23473–23479. 16375321 10.1021/jp0542266

[11] Mao Y, Wang X, Xia S, et al. High-voltage charging-induced strain, heterogeneity, and micro-cracks in secondary particles of a nickel-rich layered cathode material. Adv Funct Mater. 2019 5 1;29(18):1900247.

[12] Jung R, Metzger M, Maglia F, et al. Oxygen release and its effect on the cycling stability of LiNi_x_Mn_y_Co_z_O_2_ (NMC) cathode materials for li-ion batteries. J Electrochem Soc. 2017;164(7):A1361–77.

[13] Hwang BJ, Tsai YW, Carlier D, et al. A Combined Computational/Experimental Study on LiNi_1/3_Co_1/3_Mn_1/3_O_2_. Chem Mater. 2003;15(19):3676–3682.

[14] Radin MD, Hy S, Sina M, et al. Narrowing the gap between theoretical and practical capacities in Li-ion layered oxide cathode materials. Adv Energy Mater. 2017;7(20):1–33.

[15] Peng L, Zhu Y, Khakoo U, et al. Self-assembled LiNi_1/3_Co_1/3_Mn_1/3_O_2_ nanosheet cathodes with tunable rate capability. Nano Energy. 2015;17:36–42.

[16] Gao P, Yang G, Liu H, et al. Lithium diffusion behavior and improved high rate capacity of LiNi_1/3_Co_1/3_Mn_1/3_O_2_ as cathode material for lithium batteries. Solid State Ion. 2012;207:50–56.

[17] Shaju KM, Subba Rao GV, Chowdari BVR. EIS and GITT studies on oxide cathodes, O2-Li_(2/3)+x_(Co_0.15_Mn_0.85_)O_2_ (x = 0 and 1/3). Electrochim Acta. 2003;48(18):2691–2703.

[18] Shafiei Sabet P, Sauer DU. Separation of predominant processes in electrochemical impedance spectra of lithium-ion batteries with nickel-manganese-cobalt cathodes. J Power Sources. 2019;425:121–129.

[19] Wang Q, Tian N, Xu K, et al. A facile method of improving the high rate cycling performance of LiNi_1/3_Co_1/3_Mn_1/3_O_2_ cathode material. J Alloys Compd. 2016;686:267–272.

[20] Ivanishchev AV, Bobrikov IA, Ivanishcheva IA, et al. Study of structural and electrochemical characteristics of LiNi_0.33_Mn_0.33_Co_0.33_O_2_ electrode at lithium content variation. J Electroanal Chem. 2018;821:140–151.

[21] Shaju KM, Subba Rao GV, Chowdari BVR. Influence of Li-ion kinetics in the cathodic performance of layered Li(Ni_1/3_Co_1/3_Mn_1/3_)O_2_. J Electrochem Soc. 2004;151(9):A1324.

[22] Genieser R, Ferrari S, Loveridge M, et al. Lithium ion batteries (NMC/graphite) cycling at 80 °C: different electrolytes and related degradation mechanism. J Power Sources. 2018;373:172–183.

[23] Jiang K-C, Xin S, Lee J-S, et al. Improved kinetics of LiNi_1/3_Mn_1/3_Co_1/3_O_2_ cathode material through reduced graphene oxide networks. Phys Chem Chem Phys. 2012;14(8):2934–2939. 22274568 10.1039/c2cp23363k

[24] Bobrikov IA, Samoylova NY, Sumnikov SV, et al. In-situ time-of-flight neutron diffraction study of the structure evolution of electrode materials in a commercial battery with LiNi_0.8_Co_0.15_Al_0.05_O_2_ cathode. J Power Sources. 2017;372:74–81.

[25] Dolotko O, Senyshyn A, Mühlbauer MJ, et al. Understanding structural changes in NMC Li-ion cells by in situ neutron diffraction. J Power Sources. 2014;255:197–203.

[26] Lu Z, Dahn JR. Understanding the anomalous capacity of Li/Li[Ni_x_Li _(1/3−2x/3)_Mn_2/3−x/3_O_2_ cells using in situ X-Ray diffraction and electrochemical studies. J Electrochem Soc. 2002;149(7):A815.

[27] Mohanty D, Kalnaus S, Meisner RA, et al. Structural transformation of a lithium-rich Li_1.2_Co_0.1_Mn_0.55_Ni_0.15_O_2_ cathode during high voltage cycling resolved by in situ X-ray diffraction. J Power Sources. 2013;229:239–248.

[28] Tsai YW, Hwang BJ, Ceder G, et al. In-Situ x-ray absorption spectroscopic study on variation of electronic transitions and local structure of LiNi1/3Co1/3Mn1/3O2 cathode material during electrochemical cycling. Chem Mater. 2005 6 1;17(12):3191–3199.

[29] Buchberger I, Seidlmayer S, Pokharel A, et al. Aging analysis of graphite/LiNi_1/3_Mn_1/3_Co_1/3_O_2_ cells using XRD, PGAA, and AC impedance. J Electrochem Soc. 2015;162(14):A2737–46.

[30] Zhang L, Wang X, Noguchi H, et al. Electrochemical and ex situ XRD investigations on (1−x)LiNiO_2_·xLi_2_TiO_3_ (0.05≤x≤0.5). Electrochim Acta. 2004;49(20):3305–3311.

[31] Kong D, Zhang M, Xiao Y, et al. Insights into the structural evolution and Li/O loss in high-Ni layered oxide cathodes. Nano Energy. 2019;59:327–335.

[32] Weppner W, Huggins RA. Determination of the kinetic parameters of mixed-conducting electrodes and application to the system Li_3_Sb. J Electrochem Soc. 1977;124(10):1569.

[33] Larson AC, Von Dreele RB. Report No. LAUR 86-748. Program GSAS for Windows. Version 15-04-04. Los Alamos National Laboratory, New Mexico, USA; 1987.

[34] Toby BH. *EXPGUI*, a graphical user interface for *GSAS*. J Appl Crystallogr. 2001 4;34(2):210–213.

[35] Yin S-C, Rho Y-H, Swainson I, et al. X-ray/neutron diffraction and electrochemical studies of lithium Li_1-x_Co_1/3_Ni_1/3_Mn_1/3_O_2_ (x=0->1). Chem Mater. 2006;18(7):1901–1910.

[36] Paulsen JM, Larcher D, Dahn JR. O2 structure Li_2/3_[Ni_1/3_Mn_2/3_]O_2_: a new layered cathode material for rechargeable lithium batteries III. Ion Exchange. J Electrochem Soc. 2000;147(8):2862.

[37] Babu B, Shaijumon MM. Studies on kinetics and diffusion characteristics of lithium ions in TiNb_2_O_7_. Electrochim Acta. 2020;345:136208.

[38] Montoro LA, Rosolen JM. The role of structural and electronic alterations on the lithium diffusion in Li_x_Co_0.5_Ni_0.5_O_2_. Electrochim Acta. 2004;49(19):3243–3249.

[39] Yabuuchi N, Ohzuku T. Novel lithium insertion material of LiCo_1/3_Ni_1/3_Mn_1/3_O_2_ for advanced lithium-ion batteries. J Power Sources. 2003 6 1;119-121:171–174

[40] Shimoda K, Oishi M, Matsunaga T, et al. Direct observation of layered-to-spinel phase transformation in Li_2_MnO_3_ and the spinel structure stabilised after the activation process. J Mater Chem A. 2017;5(14):6695–6707.

[41] Qian K, Li Y, He Y-B, et al. Abuse tolerance behavior of layered oxide-based Li-ion battery during overcharge and over-discharge. RSC Adv. 2016;6(80):76897–76904.

[42] Xia D, Zheng J, Wang C, et al. Designing principle for Ni-rich cathode materials with high energy density for practical applications. Nano Energy. 2018 Apr 1;49:434–452.

[43] Belharouak I, Sun Y-K, Liu J, et al. Li(Ni_1/3_Co_1/3_Mn_1/3_)O_2_ as a suitable cathode for high power applications. J Power Sources. 2003;123(2):247–252.

[44] Arroyo Y de Dompablo ME, Ceder G. On the origin of the monoclinic distortion in Li_x_NiO_2_. Chem Mater. 2003 1 1;15(1):63–67.

[45] Yoon W, Chung K, McBreen J, et al. Study on structural changes of LiCo_1/3_Ni_1/3_Mn_1/3_O_2_ and LiNi_0.8_Co_0.15_Al_0.05_O_2_ during first charge using in situ XRD. 2006.

[46] Shannon RD. Revised effective ionic radii and systematic studies of interatomic distances in halides and chalcogenides. Acta Crystallogr Sect A. 1976 9 1;32(5):751–767.

[47] Labrini M, Saadoune I, Almaggoussi A, et al. The Li_y_Ni_0.2_Mn_0.2_Co_0.6_O_2_ electrode materials: a structural and magnetic study. Mater Res Bull. 2012 4 1;47:1004–1009.

[48] Choi J, Manthiram A. Comparison of the electrochemical behaviors of stoichiometric LiNi_1/3_Co_1/3_Mn_1/3_O_2_ and lithium excess Li _1.03_(Ni_1/3_Co_1/3_Mn_1/3_)_0.97_O_2_. Electrochem Solid-State Lett. 2004;7(10):A365.

[49] Li W, Reimers JN, Dahn JR. In situ x-ray diffraction and electrochemical studies of Li_1-x_NiO_2_. Solid State Ion. 1993;67(1–2):123–130.

[50] Gabrisch H, Yazami R, Fultz B. Hexagonal to Cubic spinel transformation in lithiated cobalt oxide. J Electrochem Soc. 2004;151(6):A891.

[51] Reimers J, Dahn J. Electrochemical and in situ X-Ray diffraction studies of lithium intercalation in Li_x_CoO_2_. J Electrochem Soc. 1992 8 1;139:2091–2097

[52] Liu Z, Yu A, Lee JY. Synthesis and characterization of LiNi_1−x−y_Co_x_Mn_y_O_2_ as the cathode materials of secondary lithium batteries. J Power Sources. 1999;81–82:416–419.

[53] Arai H, Okada S, Ohtsuka H, et al. Characterization and cathode performance of Li_1−x_Ni_1+x_O_2_ prepared with the excess lithium method. Solid State Ion. 1995;80(3):261–269.

[54] Kalyani P, Kalaiselvi N. Various aspects of LiNiO_2_ chemistry: a review. Sci Technol Adv Mater. 2005;6(6):689–703.

