# Supplementary material for: Determining phase transitions of layered oxides via electrochemical and crystallographic analysis
Source: Sci Technol Adv Mater. 2020 Sep 15;21(1):653–60. doi: 10.1080/14686996.2020.1814116 (PMC7534273; doi:10.1080/14686996.2020.1814116)
Supplement: Supplemental Material [file TSTA_A_1814116_SM7890.pdf]

Supplementary Information

Determining phase transitions of layered oxides via electrochemical and crystallographic analysis

Katja Fröhlich[a], Isaac Abrahams[b], Marcus Jahn[a]

[a]*Electric Drive Technology, AIT Austrian Institute of Technology GmbH, Vienna, Austria;*

[b]*School of Biological and Chemical Sciences, Queen Mary University of London, London, United Kingdom.*

Table S1: Crystallographic parameters for NMC

Parameter	Value
**Crystal system**	Hexagonal
**Space group**	*R*-3*m*
***a* (Å)**	2.86
***c* (Å)**	14.227

Table S2: Refined structural parameters for synthesized NMC. Estimated standard deviations are given in parentheses

Name	Lithium nickel manganese cobalt oxide
**Formula**	$LiNi_{1/3}Mn_{1/3}Co_{1/3}O_2$
**Formula weight**	96.46 g mol^{-1}
**Crystal system**	Hexagonal
**Space group**	*R*-3*m*
**Unit cell dimensions**	a = 2.8634(1), c = 14.242(1) Å
**Volume**	101.12(1) $Å^3$
**Z**	3
**Density (calculated)**	4.754 g cm^{-3}
**2θ range refined**	5-119°
**No. of observations/restraints/parameters**	344/0/23
**Total no. of reflections used**	64
**Peak shape**	Pseudo-Voigt
**Final R-factors**	R_p = 0.0181, R_{wp} = 0.0229<br>R_{ex} = 0.0219, χ^2 = 1.0934

Table S3: Atomic coordinates and isotropic thermal parameters for NMC powder. Estimated standard deviations are given in parentheses.

Atom	Site	*x*	*y*	*z*	Occ.	U_{iso} ($Å^2$)
**Li**	3b	0	0	0.5000	0.9794	0.012(7)
**Ni**	3b	0	0	0.5000	0.0207	0.012(7)
**Li**	3a	0	0	0	0.0207	0.024(1)
**Ni**	3a	0	0	0	0.3133	0.024(1)
**Mn**	3a	0	0	0	0.3333	0.024(1)
**Co**	3a	0	0	0	0.3333	0.024(1)
**O**	6c	0	0	0.2589(3)	1	0.024(2)

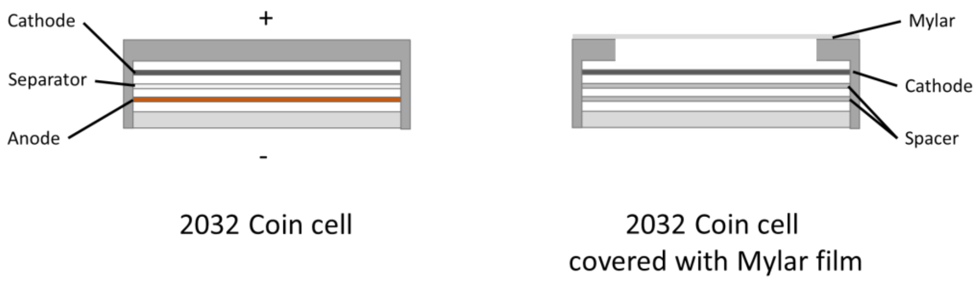

Fig. S1: Schematic of the type 2032 coin cell covered with Mylar foil (right), compared to a usual coin cell (left).

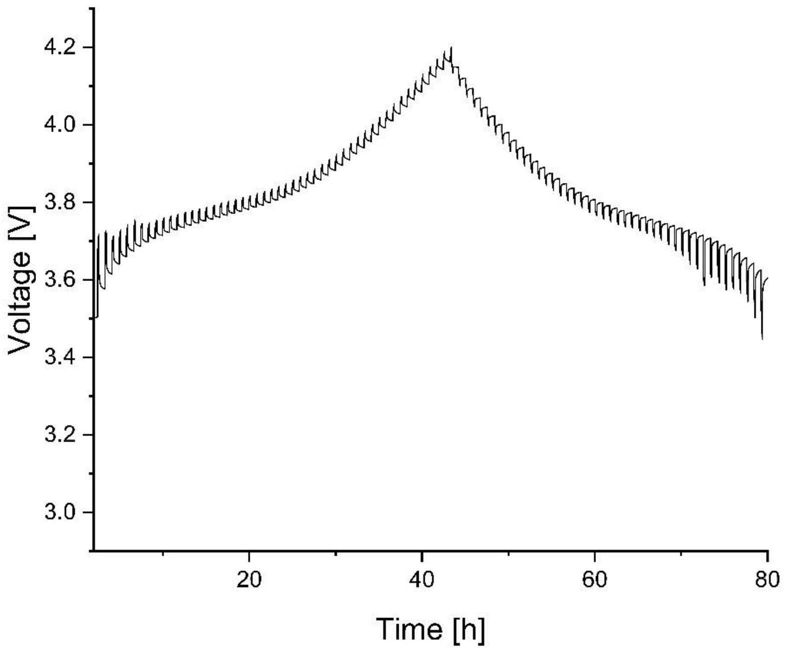

Fig. S2: Measured GITT curve over the whole cycling range. Ranges with no straight-line behaviour of the voltage curve during the pulse were not considered in the calculation of $\widetilde{D}$.

To determine the lithium ion diffusion coefficient D_{Li+}, the Wagner or thermodynamic factor was estimated via (1,2):

$$\Phi = -\left(\frac{xF}{RT}\right)\left(\frac{dU}{dx}\right)$$

where F represents the Faraday constant, R the universal gas constant, T the absolute temperature and x the moles Li^{+} in the NMC structure. For estimation of the thermodynamic or Wagner factor, the differentials dU/dx were replaced by the differences between the data points ΔU and Δx. The component diffusion coefficient can then be calculated via the relation:

$$\widetilde{D} = \Phi \cdot D_{Li+}$$

1. Weppner, W., Huggins RA. Determination of the Kinetic Parameters of Mixed-Conducting Electrodes and Application to the System Li3Sb. J Electrochem Soc. 1977;124(10):1569.
2. Wagner C. Beitrag zur Theorie des Anlaufvorgangs. Zeitschrift für Phys Chemie. 1933;21B(1):25–41.

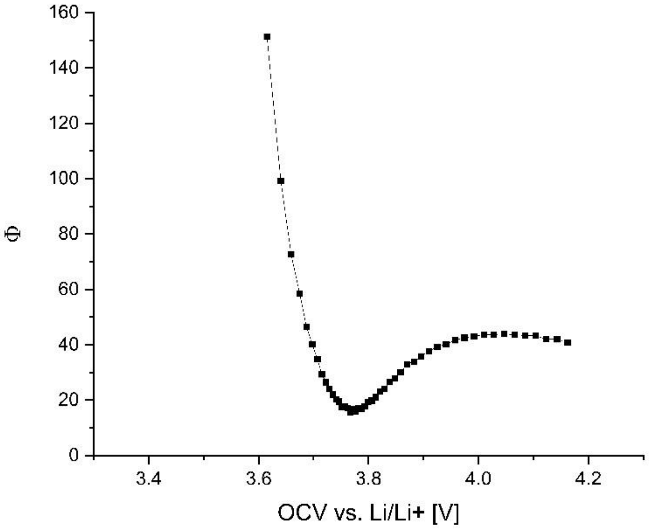

a)

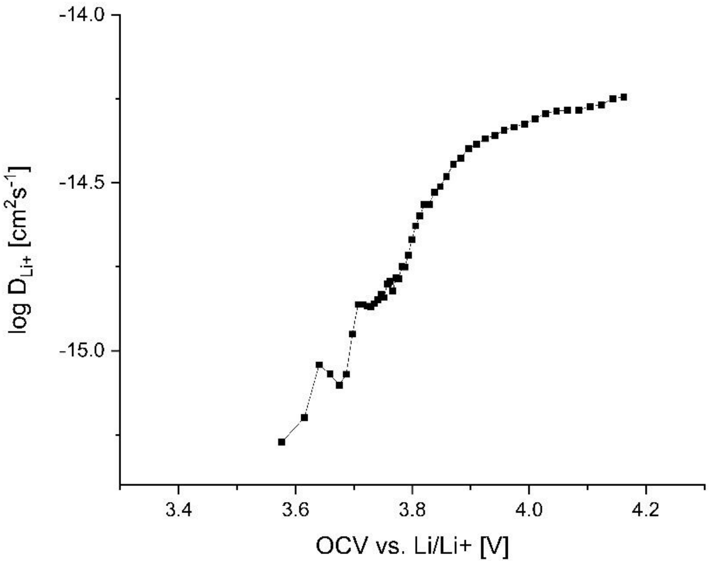

b)

Fig. S3: (a) Variation of thermodynamic factor and (b) component diffusion coefficient, D_{Li+}, with OCV as derived from the GITT measurement.

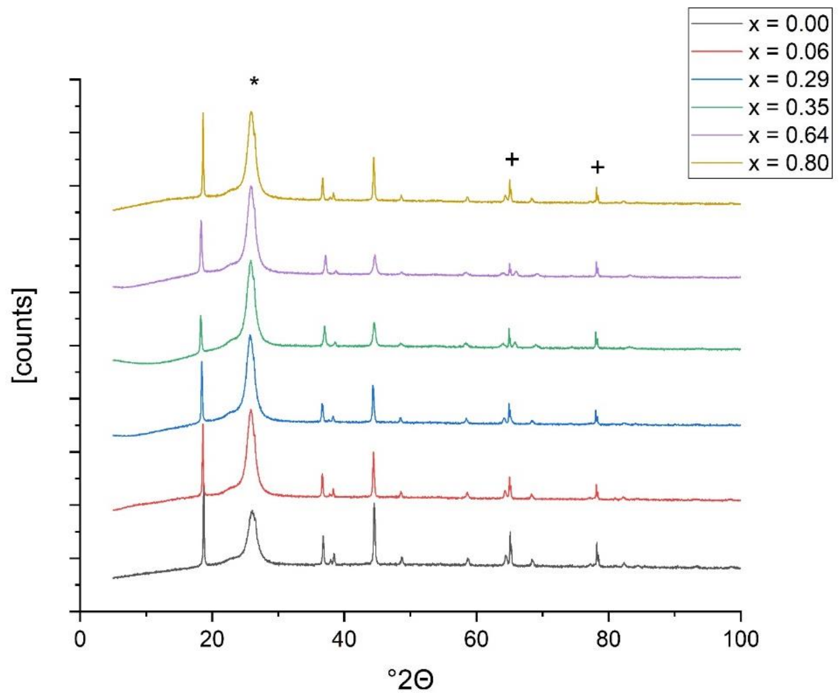

Fig. S4: Diffraction profiles for $Li_{1-x}Ni_{1/3}Mn_{1/3}Co_{1/3}O_2$ cathodes (* mylar foil, + aluminium current collector).

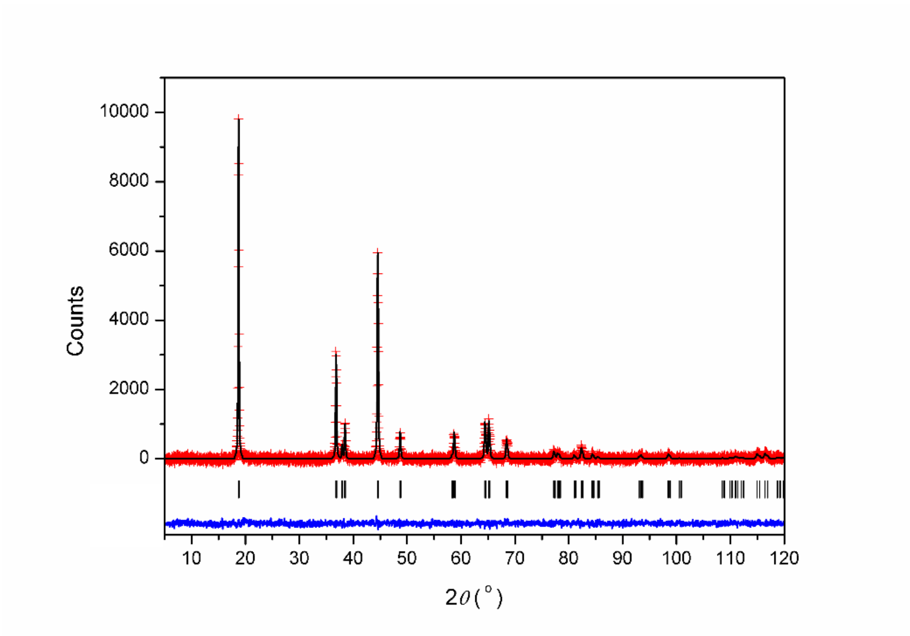

Fig. S5: Fitted diffraction profile (background subtracted) for synthesized NMC powder, showing observed (+ symbols) calculated (line) and difference (lower) profiles. Reflection positions are indicated by markers.
